# Among the Colleges

**Published:** 1899-09

**Authors:** 


### AMONG THE COLLEGES.

After an earnest effort for a number of years to maintain a Veterinary Department in connection with the Detroit College of Medicine the trustees have decided to close the same.

Many changes from deaths, resignations, etc., have followed one after another in the career of this institution, and, ever desirous of maintaining an advanced curriculum, the school has had a severe struggle for existence. The nearby competition of long-established two-year schools was an added factor in thwarting the progress of this school, while the recently enacted law in Michigan establishing a two-years' standard for her graduates was a final blow, which, no doubt, largely determined the trustees in their decision to no longer make an effort to maintain the institution. The JOURNAL regrets very much to have to note these stern facts; but these are strange times, and the tendency to amalgamation and the building up of a few strong institutions in our land in certain scientific pursuits is an evolution coming as sure as that now going on in the commercial world.

---

HARTLEY (*Annals of Surgery*) proposes the following classification of tumors based upon the exciting cause: (1) As a result of traumatism. (2) As the result of inflammatory processes, especially those followed by cicatrization and ulceration—*i.e.*, a local disturbance in the nutrition of a part. (3) As the result of congenital anomalies. (4) As the result of disturbance in nutrition, due to toxins, chemical or possibly parasitic, developed most frequently upon a soil prepared by traumatism, inflammation, or a sequestral anomaly.

